# Inter-observer reliability of alternative diagnostic methods for proximal humerus fractures: a comparison between attending surgeons and orthopedic residents in training

**DOI:** 10.1186/s13037-019-0195-3

**Published:** 2019-03-11

**Authors:** Luiz Fernando Cocco, João Alberto Yazzigi, Eduardo Felipe Kin Ito Kawakami, Helio Jorge Fernandes Alvachian, Fernando Baldy dos Reis, Marcus Vinicius Malheiro Luzo

**Affiliations:** 1 Department of Orthopedics and Traumatology, (DOT/UNIFESP)- Escola Paulista de Medicina, Universidade Federal de São Paulo, Rua Napoleão de Barros, 715, 01 andar, São Paulo, SP CEP 04024-002 Brazil; 2 Department of Orthopedics and Traumatology, UNIFESP, São Paulo, Brazil; 3 Department of Diagnostic Imaging, UNIFESP, São Paulo, Brazil

**Keywords:** 3D models, Augmented reality, Holographies, Humerus fracture

## Abstract

**Background:**

Proximal humerus fractures are frequent, and several studies show low diagnostic agreement among the observers, as well as an inaccurate classification of these lesions. The divergences are generally correlated with the experience of the surgeons as well as the diagnostic methods used. This paper challenges these problems including alternative diagnostic methods such as 3D models and augmented reality (holography) and including the observers’ period of medical experience as a factor.

**Methods:**

Twenty orthopedists (ten experts in shoulder surgery and ten experts in traumatology) and thirty resident physicians in orthopedics classified nine proximal humerus fractures randomly distributed as x-ray, tomographies, 3D models and holography, using AO/ASIF and Neer’s classification. In the end, we evaluated the intra- and inter-observer agreement between diagnostic methods and whether the experience of the observers interfered in the evaluations and the classifications used.

**Results:**

We found overall kappa coefficients ranging from 0.241 (fair) to 0.624 (substantial) between the two classifications (AO / ASIF and Neer), concerning the diagnostic methods used. We identified image modality differences (*p* = 0.017), where 3D models presented an average kappa coefficient value superior to that of tomographies. There were no differences between kappa scores for x-ray and holography compared to the others. The kappa scores for AO / ASIF classification and Neer classification and subdivided by observer period of experience showed no differences concerning the diagnostic method used.

**Conclusions:**

3D models can substantially improve diagnostic agreement for proximal humerus fractures evaluation among experts or resident physicians. The holography showed good agreement between the experts and can be a similar option to x-ray and tomography in the evaluation and classification of these fractures. The observers’ period of experience did not improve the diagnostic agreement between the image modalities studied.

**Trial registration:**

Registered in the Brazil Platform under no. CAAE 88912318.1.0000.5487.

## Background

Proximal humerus fractures are very common, affecting a significant number of adults and elderly victims of trauma or falls and are likely to become even more prevalent with increased life expectancy and association with osteoporosis [1]. However, an accurate understanding of proximal humerus fractures, as well as its therapeutic proposal, is a source of divergence between physicians and researchers [2]. Among the main causes related to low levels of agreement are the inexperience among the professionals involved and the interpretation of the images [3–5].

Several classifications have been proposed over the years to standardize diagnoses and to guide treatment. Charles Neer in 1970 [6, 7] and the Arbeit Gemeinschaft für Osteosynthesefragen group (AO/ASIF) [8] are the best-known classification systems and widely used by specialized services of orthopedic physicians training. Nevertheless, intra- and inter-observer studies on diagnostic agreements usually show low concordance between evaluators and the classifications used [9–14].

The development of technologies and softwares capable of customized reproduction of daily objects [15, 16], introduced 3D models as a method for evaluating proximal humerus fractures, improving the understanding and the treatment schedule for some patients. Other authors have also used 3D models to understand complex fractures in the pelvis, acetabulum and tibial plateau, disseminating 3D models as a method of diagnosis and schedule of surgical treatments [17–19]. 3D models are also useful in teaching and training in the medical area. Awan et al. [20] showed the improvements in the understanding of complex acetabular fractures reproduced in 3D models and reported by medical residents.

Augmented reality or holographies are similarly proving to be useful in different areas. Even though it is still not officially considered as a diagnostic method, they show considerable potential among researchers. The symbiosis between this tool and surgical medical specialties seems irreversible. The possibilities for teaching and training resident physicians, or even specialists, support the growing number of publications on the subject [21–26].

Therefore our study aims to present the intra- and inter-observer diagnostic agreement for proximal humerus fractures, using the classifications proposed by Neer and the Group AO / ASIF, together with two diagnostic methods (3D models and augmented reality) apart from those traditionally used (x-ray and tomography). In addition, this study plans to correlate the evaluators’ period of experience in the classification of proximal humerus fractures using the four proposed imaging modalities.

## Methods

**Fig. 1:**
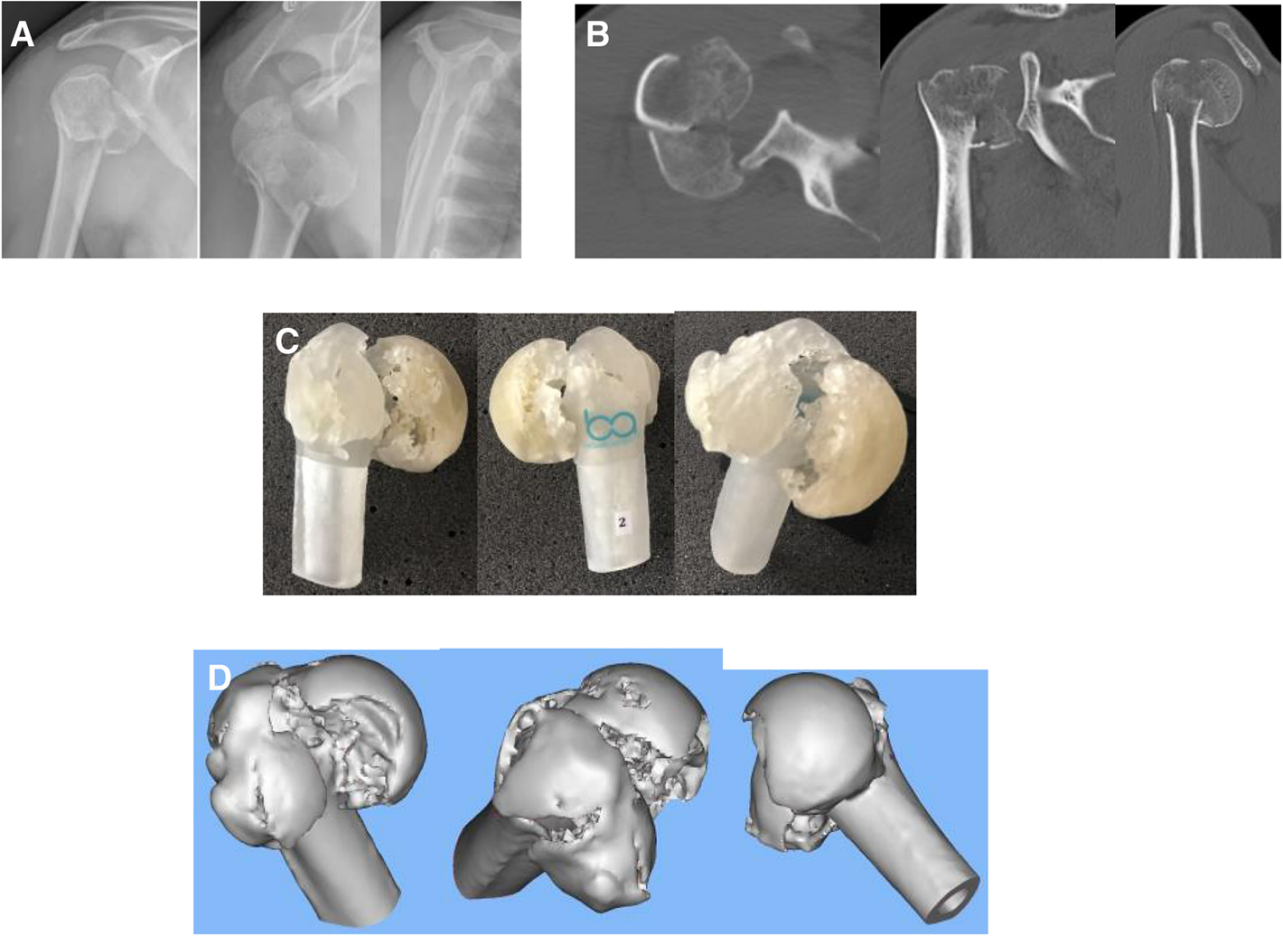
Images of the proximal fracture of the right humerus, presented in shoulder x-rays (**a**), tomography (**b**), 3D model (**c**) and augmented reality / holography (**d**) to be classified according to Neer and AO / ASIF Group classifications

This study was observational, cross-sectional with a presentation of proximal humerus fractures as digital x-rays, tomography, 3D models and augmented reality to 2 groups of doctors (1 and 2; see Fig. 1). Although each group was submitted to the four exams, the images were presented at random and in specific sessions, making it difficult to correlate any of them during the evaluations.

## Sample size determination

A sample of 9 cases was determined by statistical analysis, in order to obtain a 95% confidence interval, with amplitude of 0.40 for a kappa concordance coefficient estimated at 0.70. A standard deviation of 0.30 was assumed for calculations [27–29].

## Experimental groups

The groups were identified at the time of evaluation as follows:

- Group 1: Twenty experts in shoulder or traumatology from the Brazilian Society of Shoulder and Elbow Surgery (SBCOC) and Brazilian Society of Orthopedic Trauma (SBTO), respectively; observers period of experience - up to 5 years, between 5 and 10 years, over 10 years.
- Group 2: Thirty resident physicians in orthopedics and traumatology from Department of Orthopedics and Traumatology, UNIFESP / EPM; attending the first, second or third year of the course.

Likewise, the observers were not identified and were not exposed during the study period.

The x-ray and CT (computed tomography) images originated from the database of the Hospital Samaritano de São Paulo, Americas Medical Service, and were used for the 3D models and holography reconstruction through specific software by BioArchitects Company and donated for the study. We used the Objet350 Connex 3 printer, with a speed of 12 mm/ hour, 16 μm layers, compatible with Windows 7 and 8. The pieces were printed in resin (photopolymer), with high resolution and in real size, and takes an average of two hours and thirty minutes per model.

No patient identification information was used to guarantee confidentiality, so we request exemption from the informed consent form.

**Fig. 2:**
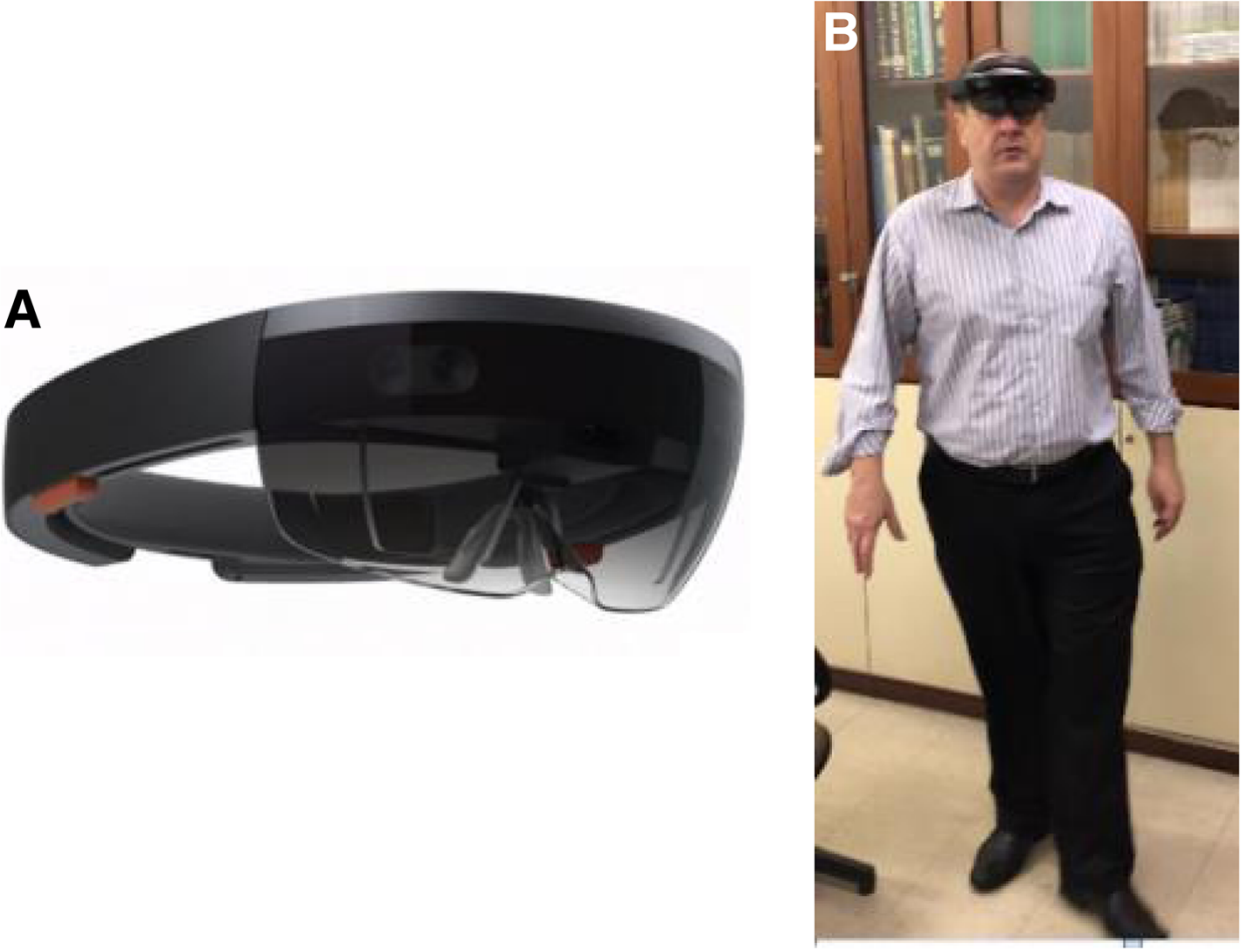
**a** Augmented reality glasses (Microsoft Hololens) used to evaluate proximal humerus fractures. **b** An orthopedist (Group 2) evaluating fractures by augmented reality / holography

In order to evaluate the proximal humerus fractures through the holographs, glasses were available (Hololens) with the proper positioning of the hologram on the lens according to the user’s viewing angle (Fig. 2a and b).

**Fig. 3:**
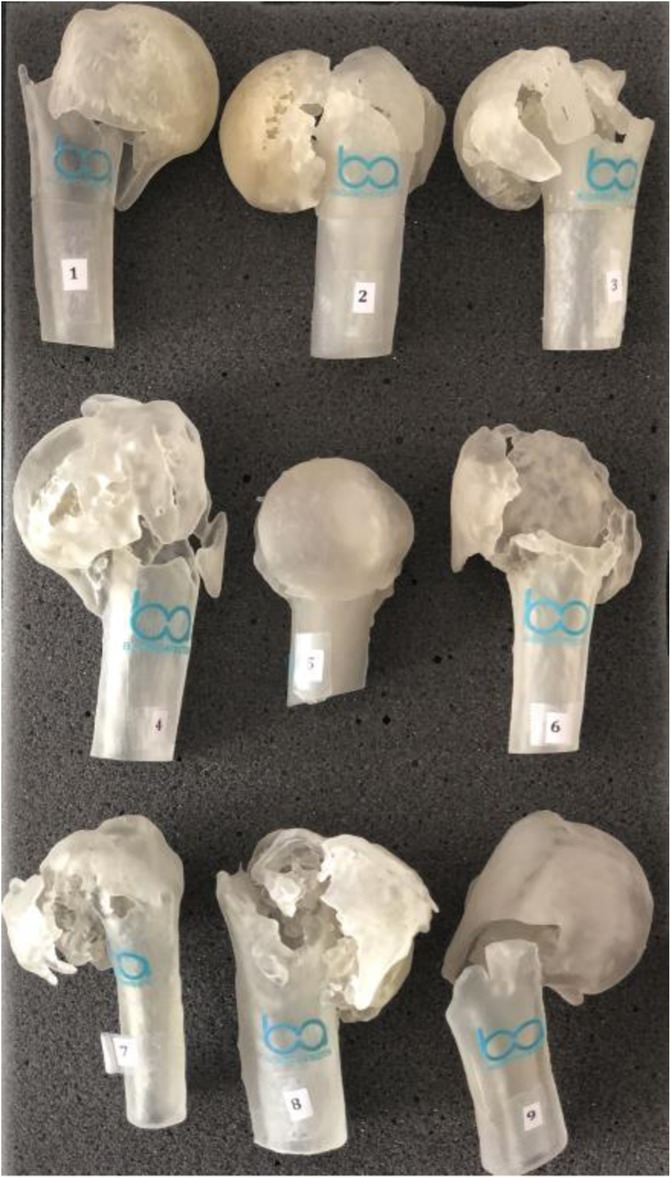
Proximal humerus fractures 3D models used for classification of fractures according to AO / ASIF Group and Neer, 1970

Biomodels are replicas of anatomical regions of patients, resulting in a three-dimensional model identical to the original. 3D model reconstruction, also known as prototyping, is the end product of this process. Each of the evaluated proximal humerus fractures went through this process, originating the models used for the assessment (Fig. 3).

The researchers selected the 9 fractures based on the quality of the radiographic images and whether they presented the complete tomographic sequences. Adults (bone growth plate closed) of both sexes were included, without restrictions on laterality. Images with suspected pathological (neoplastic) fractures, infectious diseases, previous fractures in the proximal humerus, congenital deformities or morphological alterations were not included.

Due to the absence of objective correspondence between the AO / ASIF Classification subtypes (A1.1, A1.2, A2.1, A2.2 etc) and Neer classification, we decided to use only Groups A, B and C adopted by the AO / ASIF, with correspondence to 2, 3 and 4 parts respectively, and published in the Journal of Orthopedic Trauma in 2018 (8).

**Fig. 4:**
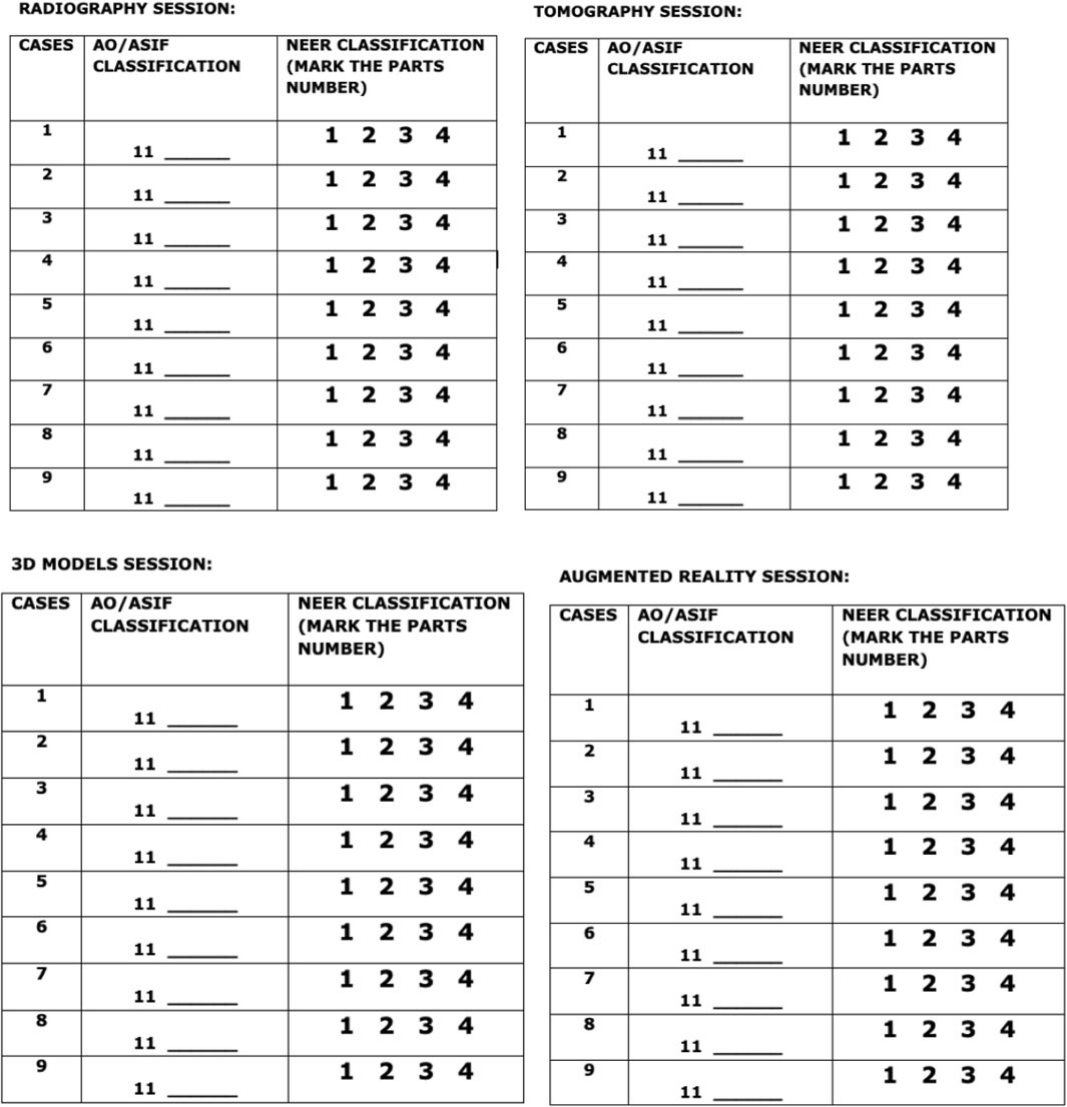
Questionnaires for the evaluation of proximal humerus fractures for the AO / ASIF and Neer Classifications, using x-rays, tomographies, 3D models and augmented reality (holography)

Therefore, we obtained the following distribution:

1. Three fractures in 02 parts (according to Neer, 1970 or 11A according to AO / ASIF);
2. Three fractures in 03 parts (according to Neer, 1970 or 11B or 11C according to AO / ASIF);
3. Three fractures in 04 parts (according to Neer, 1970 or 11C according to AO / ASIF);

During the analysis of the images and the questionnaires filling, the two groups received the AO / ASIF Group classifications and Neer (1970) as a table, which could be consulted throughout the evaluation, helping the observers choose the answers that they judged compatible with the exams presented (Fig. 4).

**Fig. 5:**
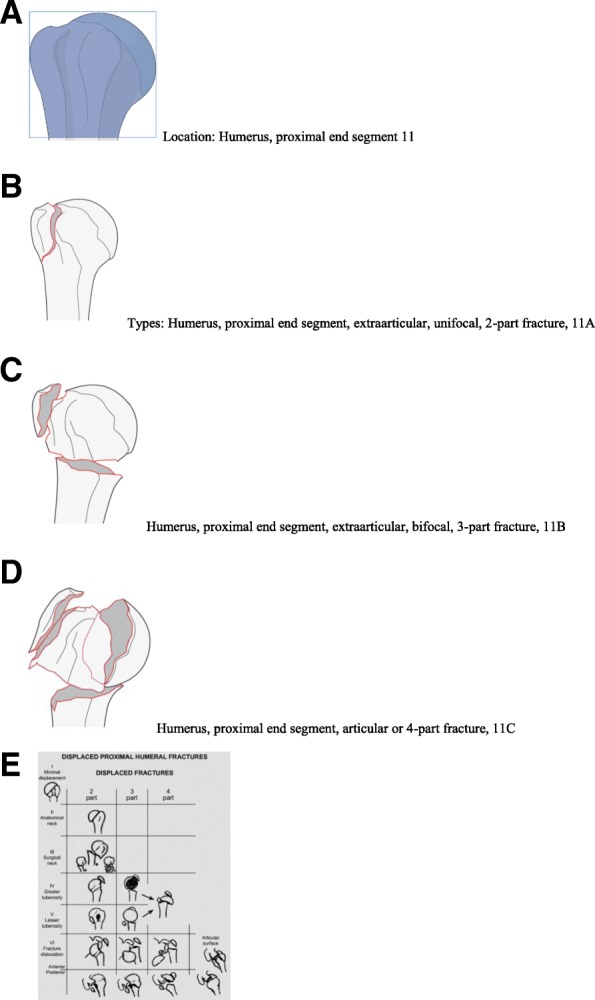
**a**, **b**, **c** and **d**: Classification table for proximal humerus fractures; Font: Kellam and Meinberg (2018); 5E Neer classification. Font: Bradley et al. (2013) [7]

Figure 5 show the classification tables for proximal humerus fractures used for the study.

### Statistical analysis

In order to evaluate the inter-observer agreement between the AO / ASIF and Neer classifications for each diagnostic method (x-ray, tomography, 3D models and augmented reality), and for each group, the overall kappa coefficients were calculated [30].

For the intra-observer evaluation for each group (between AO / ASIF and Neer classifications by diagnostic method), the kappa coefficients were calculated similarly [28]. The kappa coefficient summary values were presented as mean, quartiles, standard deviation, minimum and maximum. Additionally, differences in kappa coefficients were compared using Analysis of Variance (ANOVA) with repeated measures. When differences between the means were detected, multiple comparisons using Bonferroni were performed to identify groups of different means, maintaining the level of significance.

Comparisons of kappa coefficients by observers period of experience, year of residence and observer category (Groups 1 and 2) were performed using the Kruskal-Wallis test (small sample size), Analysis of Variance (ANOVA) and Student’s t-test, respectively. Data normality was verified by the Kolmogorov-Smirnov test.

A significance level of 5% was used for all statistical tests. Statistical analyses were performed using the statistical software SPSS 20.0 and STATA 12.

## Results

I. Inter-observer agreement among the experts (Group 1)

**Fig. 6:**
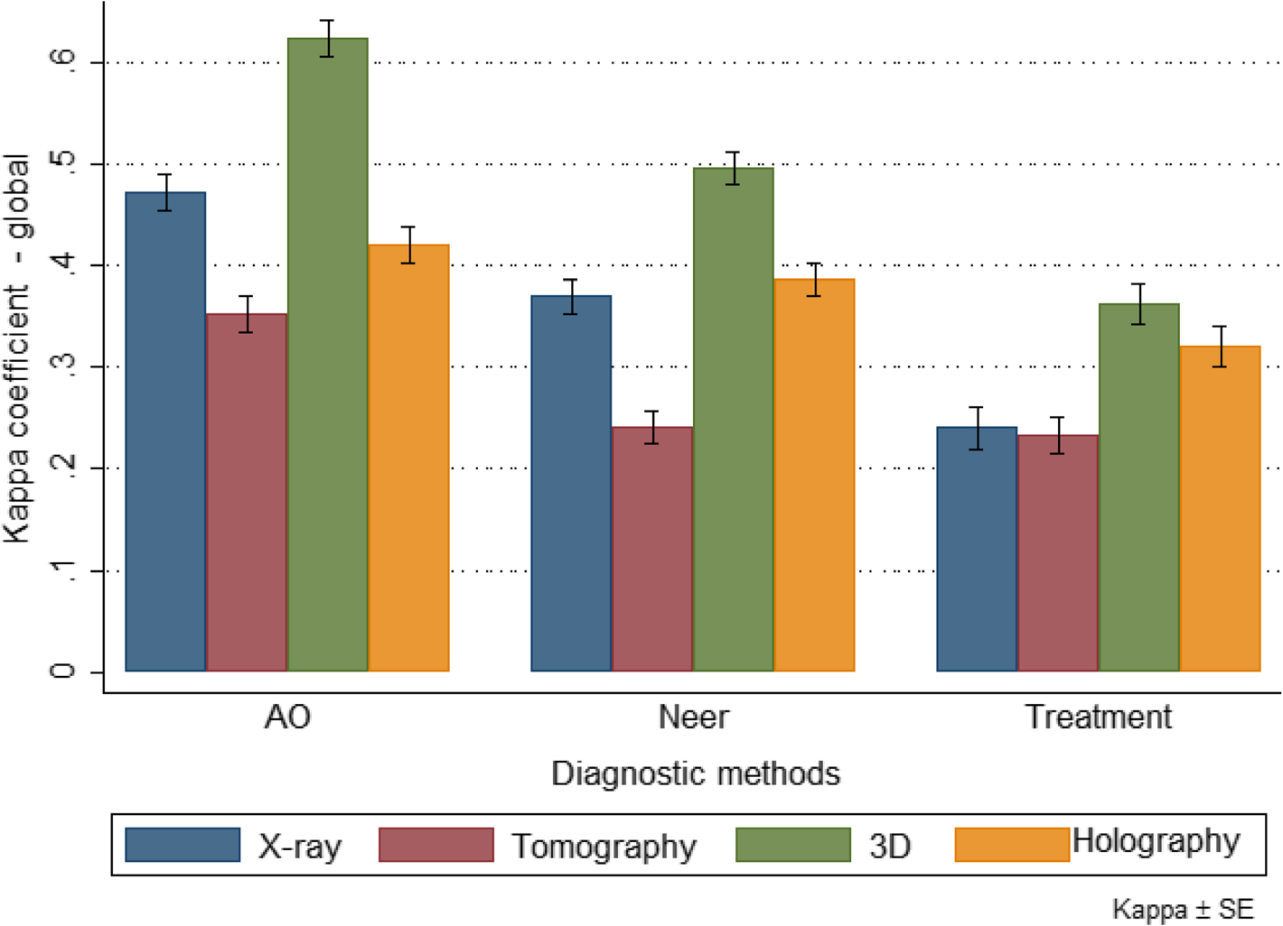
Overall kappa coefficient and standard deviation by diagnostic method and expert classification (Group 1)

Table 1 and Fig. 6 show overall kappa coefficients by expert classification and diagnostic method (Group 1). For each procedure, agreement was also evaluated by dichotomizing the type of response (each response versus the other responses).

**Table 1:** Overall kappa coefficient by diagnostic method and expert classification (Group 1)

	X-ray		Tomography		3D models		Augmented Reality/Holography	
	Kappa	p	Kappa	p	Kappa	p	Kappa	p
AO	0.472	<0.001	0.352	<0.001	0.624	<0.001	0.421	<0.001
A^a^	0.561	< 0.001	0.397	< 0.001	0.711	< 0.001	0.520	< 0.001
B^a^	0.099	< 0.001	0.075	< 0.001	0.251	< 0.001	0.061	0.006
C^a^	0.628	< 0.001	0.520	< 0.001	0.763	< 0.001	0.555	< 0.001
Neer	0.371	<0.001	0.241	<0.001	0.497	<0.001	0.387	<0.001
1 part	0.019	0.219	0.020	0.205	0.058	0.008	0.139	< 0.001
2 parts	0.542	< 0.001	0.426	< 0.001	0.719	< 0.001	0.536	< 0.001
3 parts	0.101	< 0.001	0.042	0.040	0.225	< 0.001	0.164	< 0.001
4 parts	0.435	< 0.001	0.296	< 0.001	0.554	< 0.001	0.446	< 0.001

*AO* Arbeit Gemeinschaft für Osteosynthesefragen classification, *Neer* Charles Neer classification

^a^ Groups A, B and C adopted by the AO / ASIF, with correspondence to 2, 3 and 4 parts respectively (Kellam and Meinberg, 2018) [8]

*N* = 20 observers

We noticed that among the experts (Group 1) the overall kappa coefficients (inter-observer agreement) ranged from 0.241 (fair) to 0.624 (substantial), see Table 1. 3D models, in general, presented better kappa coefficients compared to the others. On the other hand, the tomography presented one of the smallest kappa coefficients. It was also observed that for AO / ASIF classification, type B, presented the lowest kappa coefficient and type C the largest, regardless of the diagnostic method used. In the Neer classification, however, the highest kappa coefficients were observed for fractures in 4 parts, followed by fractures in 2 parts.

**Fig. 7:**
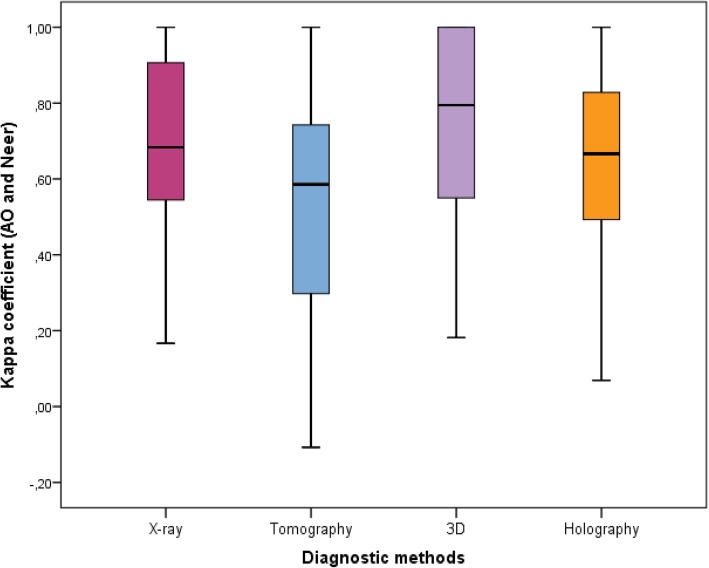
Box-Plot of kappa coefficients for intra-observers agreement between AO / ASIF classification and Neer by expert diagnostic method (Group 1). The plot shows the quartiles (1st quartile, median and 3rd quartile), minimum and maximum

According to Table 2 and Fig. 7, there was different intra-observers agreement by statistically relevant diagnostic method (*p* = 0.017) among the experts (Group 1). It was also verified that the 3D models had a mean kappa coefficient superior to tomography, whereas mean values of x-ray and holography were not different from the others. In Fig. 7, the quartiles (1st quartile, median and 3rd quartile), minimum and maximum are represented as a Box-Plot diagram.

**Table 2:** Summary of kappa coefficients for intra-observers agreement between AO / ASIF and Neer classifications by expert diagnostic method (Group 1)

Diagnostic Method	Means	Standard deviation	Minimum	Maximum	1st Quartile	Median	3rd. Quartile	N
X-ray	0.677	0.270	0.167	1.000	0.531	0.683	0.953	20
Tomo	0.531^b^	0.316	−0.108	1.000	0.280	0.586	0.778	20
3D	0.730^a^	0.265	0.182	1.000	0.525	0.795	1.000	20
Holographic	0.654	0.278	0.069	1.000	0.489	0.666	0.831	20

Effects of diagnostic method: F3,57 = 3.67 (p = 0.017)

Kolmogorov-Smirnov test for normality (*p* = 0.638)

(a) and (b) show different means according to multiple comparisons using Bonferroni adjustments

According to Table 3, there were no differences for mean values between the diagnostic methods and the period of experience among the experts (Group 1).

II. Inter-observer agreement between residents (Group 2)

**Table 3:** Summary of kappa coefficient values for agreement between AO / ASIF and Neer classifications by period of experience, according to expert diagnostic method (Group 1)

Diagnostic Method	Means	Standard Deviation	Minimum	Maximum	1st. Quartile	Median	3rd. Quartile	N	p
X-ray	0.677	0.270	0.167	1.000	0.531	0.683	0.953	20	0.930
0 to 5 years	0.686	0.274	0.182	1.000	0.488	0.683	1.000	14	
5 to 10 years	0.583	0.589	0.167	1.000	–	–	–	2	
More than 10 years	0.694	0.116	0.571	0.800	0.583	0.701	0.796	4	
Tomography	0.531	0.316	-0.108	1.000	0.280	0.586	0.778	20	0.586
0 to 5 years	0.499	0.348	−0.108	1.000	0.183	0.563	0.813	14	
5 to 10 years	0.786	0.303	0.571	1.000	–	–	–	2	
More than 10 years	0.515	0.174	0.333	0.673	0.350	0.527	0.668	4	
3D models	0.730	0.265	0.182	1.000	0.525	0.795	1.000	20	0.238
0 to 5 years	0.706	0.268	0.182	1.000	0.570	0.715	1.000	14	
5 to 10 years	1.000	0.000	1.000	1.000	–	–	–	2	
More than 10 years	0.680	0.279	0.400	1.000	0.425	0.660	0.955	4	
Holography	0.654	0.278	0.069	1.000	0.489	0.666	0.831	20	0.828
0 to 5 years	0.631	0.309	0.069	1.000	0.350	0.666	0.875	14	
5 to 10 years	0.643	0.222	0.486	0.800	–	–	–	2	
More than 10 years	0.740	0.217	0.500	1.000	0.535	0.730	0.955	4	

*p* values from Kruskal-Wallis test

**Fig. 8:**
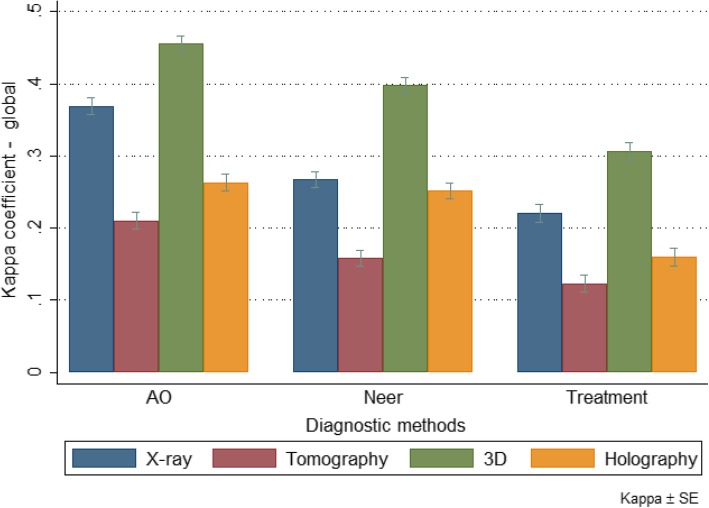
Overall Kappa coefficient by diagnostic method and classification among residents (Group 2)

Among the residents (Group 2; Table 4) the overall kappa coefficients (inter-observer agreement) ranged from 0.160 (slight) to 0.455 (moderate). It was also verified that 3D models, in general, presented greater kappa coefficients compared to the others (Table 4 and Fig. 8). On the other hand, tomography images presented one of the smallest kappa coefficients. It was also observed that in the AO / ASIF classification, type B presented the lowest kappa coefficient compared to types A and C, independent of the diagnostic method used. In the Neer classification, however, the highest kappa coefficient was observed for fractures in 2 and 4 parts, respectively.

**Table 4:** Overall kappa coefficient by diagnostic method and classification among residents (Group 2)

	RX		Tomography		3D models		Augmented Reality/Holography	
	Kappa	p	Kappa	p	Kappa	p	Kappa	p
AO	0.369	<0.001	0.210	<0.001	0.455	<0.001	0.263	<0.001
A^a^	0.470	< 0.001	0.276	< 0.001	0.613	< 0.001	0.366	< 0.001
B^a^	0.206	< 0.001	0.021	0.098	0.139	< 0.001	0.029	0,037
C^a^	0.438	< 0.001	0.335	< 0.001	0.541	< 0.001	0.341	< 0.001
Neer	0.268	<0.001	0.158	<0.001	0.397	<0.001	0.251	<0.001
1 part	0.013	0.216	0.003	0.419	0.059	< 0.001	−0.005	0.618
2 parts	0.460	< 0.001	0.230	< 0.001	0.647	< 0.001	0.432	< 0.001
3 parts	0.066	< 0.001	0.015	0.180	0.174	< 0.001	0.076	< 0.001
4 parts	0.312	< 0.001	0.257	< 0.001	0.379	< 0.001	0.250	< 0.001

*AO* Arbeit Gemeinschaft für Osteosynthesefragen classification, *Neer* Charles Neer classification

^a^ Groups A, B and C adopted by the AO / ASIF, with correspondence to 2, 3 and 4 parts respectively (Kellam and Meinberg, 2018) [8]

*N* = 30 resident physicians

**Fig. 9:**
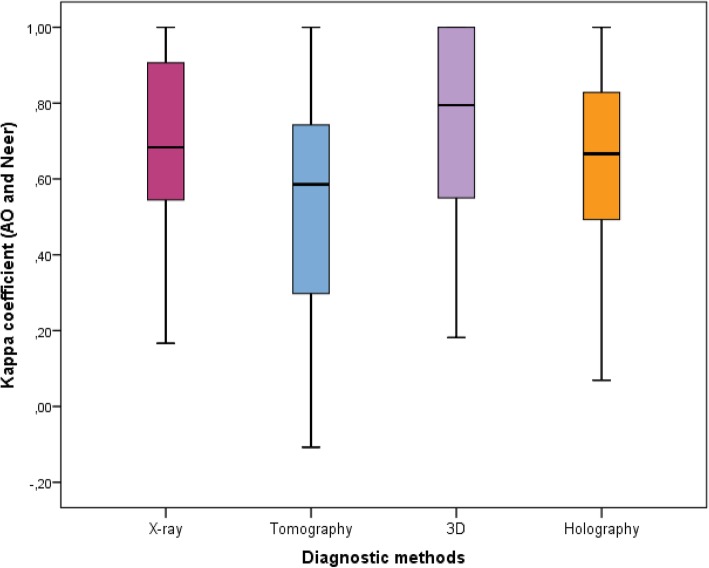
Box-Plot of kappa coefficients for intra-observer agreement between AO / ASIF and Neer classification by diagnostic method among residents (Group 2). The plot shows the quartiles (1st quartile, median and 3rd quartile), minimum and maximum

The results for intra-observers agreement are shown in Table 5 and Fig. 9. No statistically significant differences were found between diagnostic methods among residents (*p* = 0.073).

**Table 5:** Kappa coefficient summary values for intra-observer agreement between AO / ASIF and Neer classification by diagnostic method among residents (Group 2)

Diagnostic Method	Means	Standard Deviation	Minimum	Maximum	1st. Quartile	Median	3rd. Quartile	N
X-ray	0.619	0.337	−0.033	1.000	0.321	0.726	1.000	30
Tomography	0.558	0.376	−0.125	1.000	0.211	0.571	1.000	30
3D models	0.658	0.301	−0.050	1.000	0.437	0.736	0.868	30
Holography	0.671	0.295	0.087	1.000	0.490	0.675	1.000	30

Effects of diagnostic method: F3,87 = 2.41 (p = 0.073)

Kolmogorov-Smirnov test for normality (*p* = 0.348)

In relation to period of experience among residents (Table 6), there was no difference between the diagnostic methods and the residents’ period of experience (Group 2).

III. Comparing diagnostic agreement between experts and residents

**Table 6:** Summary of kappa coefficients for agreement between AO / ASIF and Neer classification by time of experience, according to diagnostic method among residents (Group 2)

Diagnostic Method	Means	Standard Deviation	Minimum	Maximum	1st. Quartile	Median	3rd. Quartile	N	p
X-ray	0.619	0.337	-0.033	1.000	0.321	0.726	1.000	30	0.689
First year	0.636	0.419	−0.033	1.000	0.218	0.824	1.000	9	
Second year	0.544	0.321	0.036	1.000	0.313	0.495	0.868	10	
Third year	0.672	0.295	0.224	1.000	0.321	0.813	0.833	11	
Tomography	0.558	0.376	-0.125	1.000	0.211	0.571	1.000	30	0.535
First year	0.608	0.396	−0.050	1.000	0.220	0.673	1.000	9	
Second year	0.446	0.380	−0.125	1.000	0.156	0.363	0.827	10	
Third year	0.618	0.369	0.045	1.000	0.237	0.633	1.000	11	
3D models	0.658	0.301	-0.050	1.000	0.437	0.736	0.868	30	0.861
First year	0.613	0.361	0.060	1.000	0.227	0.813	0.912	9	
Second year	0.690	0.301	−0.050	1.000	0.604	0.736	0.868	10	
Third year	0.666	0.273	0.321	1.000	0.400	0.640	1.000	11	
Holography	0.671	0.295	0.087	1.000	0.490	0.675	1.000	30	0.836
First year	0.695	0.369	0.087	1.000	0.333	0.833	1.000	9	
Second year	0.624	0.288	0.167	1.000	0.408	0.628	0.873	10	
Third year	0.695	0.255	0.237	1.000	0.500	0.660	1.000	11	

*p* values from ANOVA

**Fig. 10:**
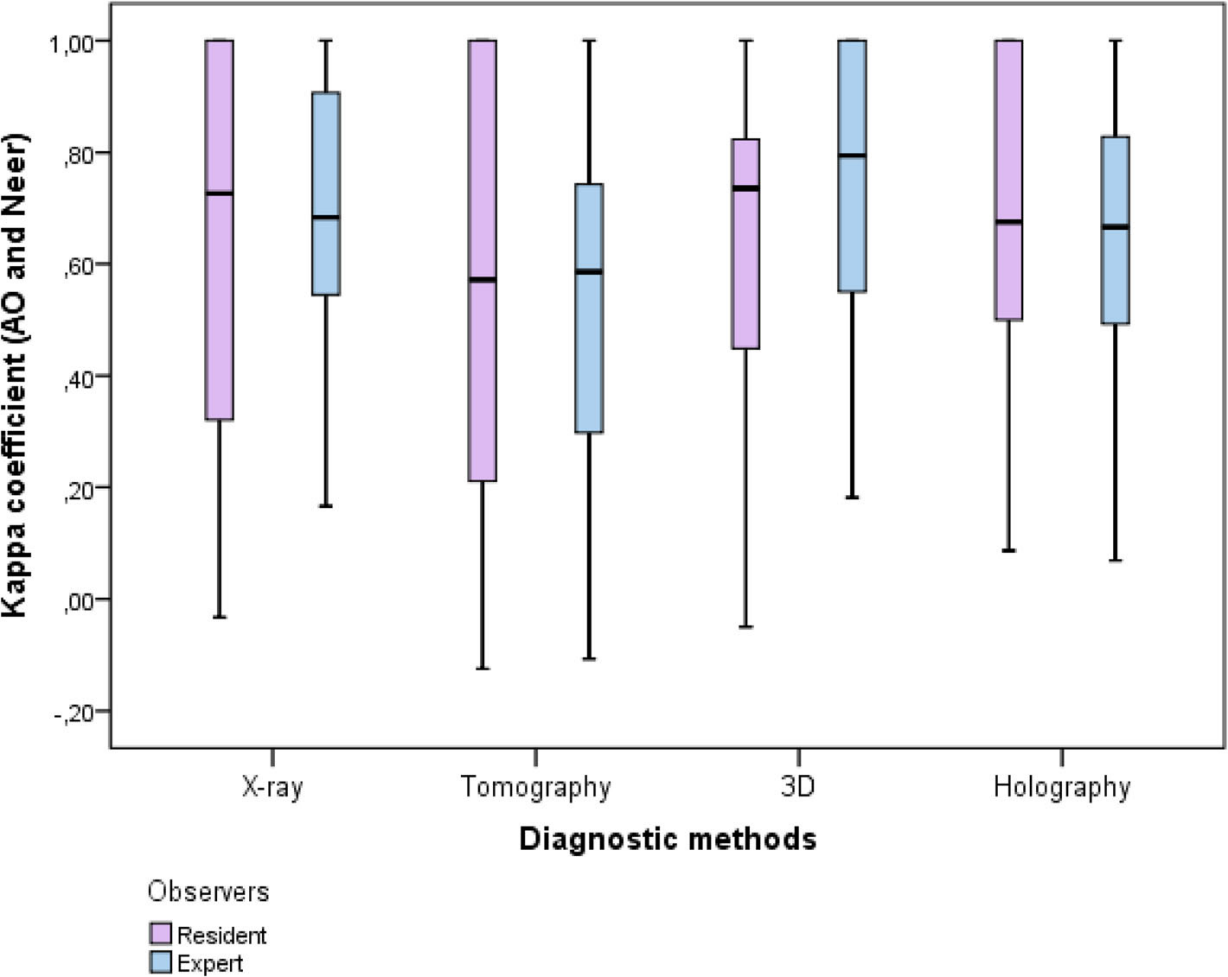
Kappa coefficients Box-Plot agreements between AO / ASIF and Neer classification by diagnostic method between experts and residents (Group 1 x Group 2). The plot shows the quartiles (1st quartile, median and 3rd quartile), minimum and maximum

According to Table 7 and Fig. 10, there were no statistically significant differences for diagnostic agreements between experts and residents (Group 1 vs. Group 2).

**Table 7:** Summary of kappa coefficients for agreement between AO / ASIF and Neer classification by period of experience, according to diagnostic method between specialists and residents (Group 1 x Group 2)

Diagnostic Method	Means	Standard Deviation	Minimum	Maximum	1st. Quartile	Median	3rd. Quartile	N	p
X-ray	0.642	0.310	-0.033	1.000	0.360	0.683	1.000	50	0.498
Resident	0.619	0.337	−0.033	1.000	0.321	0.726	1.000	30	
Expert	0.677	0.270	0.167	1.000	0.531	0.683	0.953	20	
Tomography	0.547	0.350	-0.125	1.000	0.231	0.571	0.875	50	0.794
Resident	0.558	0.376	−0.125	1.000	0.211	0.571	1.000	30	
Expert	0.531	0.316	−0.108	1.000	0.280	0.586	0.778	20	
3D models	0.687	0.287	-0.050	1.000	0.495	0.787	1.000	50	0.390
Resident	0.658	0.301	−0.050	1.000	0.437	0.736	0.868	30	
Expert	0.730	0.265	0.182	1.000	0.525	0.795	1.000	20	
Holography	0.664	0.285	0.069	1.000	0.496	0.673	1.000	50	0.836
Resident	0.671	0.295	0.087	1.000	0.490	0.675	1.000	30	
Expert	0.654	0.278	0.069	1.000	0.489	0.666	0.831	20	

*p* value - Student t-test

## Discussion

This work correlated the ability to interpret and classify proximal humerus fractures by orthopedic experts and residents using four diagnostic alternatives (x-rays, tomography, 3D models and augmented reality), through the AO / ASIF and Neer classifications (the most common ones). The agreement among the different imaging alternatives could advance the understanding and development of new diagnostic methods.

In this study, we were able to prove statistically the capacity that 3D models have for a better diagnostic agreement between the evaluators. In all analyzes and comparisons, the kappa coefficient was maintained above all the other imaging modalities. These findings can stimulate further work to be performed with different populations or higher number of cases to determine specificity and sensitivity among the imaging methods, showing possible improvements for diagnosis of proximal humerus fractures.

The augmented reality as a method of evaluation presented a significant kappa coefficient for intra-observer diagnostic agreement between the experts (Group 1), and similar to the x-rays (kappa respectively 0.654 and 0.677), but with no statistical power to differentiate both. Again, the results shown here could reinforce new studies using larger groups. Also, the interest and curiosity that the holographs generated in the observers of this study demonstrate the potential that this tool has in the processes of continued education in the medical area.

There is a large body of literature on the divergence of diagnoses that characterize proximal humerus fractures in the orthopedic routine. Perhaps the misunderstanding about this disease leads to a lack of a consensus for the treatment of patients. According to the meta-analysis of Handoll et al. [31], we still cannot conclude that surgical treatments are superior to more conservative measures. The absence of agreement persists even among the most widely accepted classification by specialists in shoulder and trauma surgery (AO / ASIF Classification Group and Neer, 1970), or the orthopedic services that train resident physicians.

In an attempt to solve or minimize such divergences, other studies [3, 4] discuss new classifications and highlight radiographic aspects (which is, after all, the most frequently used method), aiming to improve intra-observer and inter-observer agreement and standardize the diagnosis and the understanding of these fractures. Shoulder tomographies are also part of the diagnostic investigation, because they have superior sensitivity compared to x-rays for some articular fractures (head split for example), and offer greater comfort to the patients during evaluations, eliminating extreme movements (as in axillary radiographic incidence).

Although x-rays or CT scans are traditional diagnostic methods, three-dimensional models, or merely 3D models, are gaining ground as a complementary resource. Studies have shown a good acceptance by surgeons, who claim not only a better understanding of fractures, but also a facility for previous surgical programming. They refer to the intraoperative facility for placement of the implants through previous manipulation of the 3D models constructed from the initial images of the fracture [15]. Moreover, we believe that the popularization and easy access to 3D printing models can influence and change the therapeutic behavior among the surgeons. We are conducting comparative studies between treatment options and implants choice (plates, rods or prostheses) based on imaging exams and three-dimensional models. The results will be presented in future publications.

The development and feasibility of this method, however, depends on the analysis of costs and effectiveness. A 3D printing model can reach three to four times the average value of a CT or magnetic resonance imaging. The size of the pieces, the type of resin used for printing and the prototypes details directly influence the final prices. In addition, because it is new and not officially recognized as a diagnostic resource for understanding fractures in general, it could take a while until it is authorized by healthcare providers.

Another method that has been gaining ground in several areas of medical and non-medical routine is augmented reality. Also called holography, these futuristic images can provide detailed information about the fractures based on previous exams. With holographic goggles, surgeons may access fracture details that would eventually change their medical conduct [17, 21–23, 25, 26]. The beauty of the images created, as well as the novelty of the method, were reasons for considerable interest among the evaluators. The substantial diagnostic concordance compared to radiography and CT scans show the potential of its use as a diagnostic method. However, because it is an image, it was less concordant in the diagnoses when compared to 3D models. Perhaps the difference between purely visual resources (X-Ray, CT and holography) and tactile (3D models) could be related to the learning routine of surgeons. In practice, during surgeries, the manipulation of the bone fragments is complementary to the preoperative images for an understanding and performance of the surgical programming. Changes in techniques, accessibility or implants are often decided after intraoperative palpation, a measure impossible to perform by analyzing only diagnostic images. Especially in fractures of the proximal humerus, the number of fragments may be even more challenging to characterize exclusively by imaging tests. In this study, the lowest diagnostic concordance occurred in 3 parts fractures according to Neer classification, probably related to the difficulty of interpretation between the involvement of one or two tubercles, as well as the contact between the tubercles and the other parts of the fracture. The manipulation and prior visualization of 3D models for fractures of the proximal humerus can reduce problems such as this in the routine of the surgeons. Nevertheless, the augmented reality provoked considerable interest in the evaluators, motivating us for future and exciting projects in this area.

## Conclusions

3D models are suggested as a potential imaging method to improve diagnostic agreement for the evaluation of proximal humerus fractures for experts or resident physicians. The augmented reality presents a substantial diagnostic agreement between the experts and could be a similar option to x-ray and tomography in the evaluation and classification of proximal humerus fractures. The observers’ period of medical experience did not increase the diagnostic agreement between the proposed methods.

## Abbreviations

AO/ASIF: Arbeit Gemeinschaft für Osteosynthesefragen group
CT: Computed tomography
UNIFESP: Universidade Federal de São Paulo
